# Water volumes and mulches affect plant growth, leaf nutrient status and orchard soil mineral content of sweet orange cv. Mosambi

**DOI:** 10.1038/s41598-024-73262-6

**Published:** 2024-10-13

**Authors:** Kalpana Choudhary, J. Singh, N. K. Meena, Nadhir Al-Ansari, Sonali Choudhary, Ravindra Kumar Tiwari, Mahendra Choudhary, Dinesh Kumar Vishwakarma, Salah El-Hendawy, Mohamed A. Mattar

**Affiliations:** 1 Department of Fruit Science, College of Horticulture and Forestry, Jhalawar, Agriculture University, Kota, Rajasthan 324001 India; 2 Department of Civil, Environmental and Natural Resources Engineering, Lulea University of Technology, Lulea, 97187 Sweden; 3 Department of Horticulture, Sri Karan Narendra Agriculture University, Jobner, Rajasthan 303329 India; 4 School of Agriculture, Lovely Professional University, Phagwara, Punjab 144001 India; 5 Department of Agronomy, G. B. Pant University of Agriculture and Technology, Pantnagar, Uttarakhand 263145 India; 6 Department of Irrigation and Drainage Engineering, G.B. Pant University of Agriculture and Technology, Pantnagar, Uttarakhand 263145 India; 7 Department of Plant Production, College of Food and Agricultural Sciences, King Saud University, P.O. Box 2460, Riyadh, 11451 Saudi Arabia; 8 Department of Agricultural Engineering, College of Food and Agriculture Sciences, King Saud University, P.O. Box 2460, Riyadh, 11451 Saudi Arabia; 9 Krishi Vigyan Kendera, Agriculture University, Jodhpur, Rajasthan 342304 India

**Keywords:** Water volumes, Mulches, Leaf nutrients, Plant growth, Sweet orange, Plant stress responses, Agroecology

## Abstract

Day-by-day increasing irrigation water scarcity requires the application of water-saving irrigation techniques to sustain agriculture production. A two-year field investigation was conducted during 2018 to 2020 to determine the effects of various mulches and irrigation volumes on the growth, leaf chemicals and soil properties of one-year-old sweet oranges (*Citrus sinensis*) cv. Mosambi. The study included three irrigation schedules, viz.100% ET_c_ (I_1_), 80% ET_c_ (I_2_), and 60% ET_c_ (I_3_), and five different mulches were used, viz. without mulch, white polythene, coriander straw, dry grass and black polythene mulches, replicated thrice. Results demonstrated that drip irrigation with 100% ET_c_ and mulching with black polythene mulch significantly increase the plant growth attributes like height of the plant (28.64%) (30.31%), rootstock girth (36.61%) (37.90%), plant canopy spread (E-W and N-S) (EW- 63.82%, NS- 63.87%) (EW- 67.56%, NS- 67.90%) and leaf area (2.4%) (2.34%). Furthermore, plant leaf chlorophyll content (2.41 mg g^-1^) (2.41 mg g^-1^) and leaf mineral content such as N (2.39%) (2.40%), P (0.16%) (0.165%), K (1.57%) (1.59%), Ca (47.34 g kg^-1^) (47.80 g kg^-1^), Mg (4.54 g kg^-1^) (4.57 g kg^-1^), Fe (120.51 g kg^-1^) (123.15 g kg^-1^) and Zn (39.00 g kg^-1^) (37.84 g kg^-1^) were noted to be significantly (*p* ≤ 0.05) higher in plants that received 100% (were ET_c_ (I_1_) and mulching with black polythene mulch (M_1_) treatment. Taken together, the results suggested that treatments I_1_ and M_1_ have the potential to maximize plant growth, leaf chemicals and soil nutrients of sweet orange (*Citrus sinensis*) cv. Mosambi plants.

## Introduction

Rutaceae family of angiosperms is recognized for nutritionally and economically important genus *Citrus*^1^. This is the biggest family in Sapindales order, consisting of around 162 genera and 2085 plant species, dispersed across the globe^2,3^. Globally, the most grown species of this genus are mandarin, sour orange, sweet orange, lemon, lime, pummelo and grapefruit^4^ producing 135.76 MT fruits from 9.68 million hectares area^5^. At present the major citrus fruit production of India stands at 148.10 million tonnes, out of which 42.46% share relates to mandarin, 21.36% to sweet orange, 28.04% to lime and lemon and 8.13% to other citrus fruit crops (NHB, 2022). Amid *Citrus* fruits, sweet orange (*Citrus sinensis*) commonly known as tight skin orange is the second important crop in terms of production as well as cultivated area in India^6^. Brazil, China, India, USA, Mexico, Spain, Egypt are important sweet orange producing countries where it finds dominating acreage in cultivation^5^. Sweet orange is grown over 2.34 lakh hectare area with production of 4.38 million tonnes fruits per annum in India^7^. The crop covers maximum cultivated area in Andhra Pradesh, Maharashtra and Karnataka^7^.

As the plant is a perennial evergreen fruit crop of tropical and sub-tropical regions it requires water round of the year. Now a days, more than ever, the lack of water for agriculture is a major environmental impact on fruit crops, restricting the sustainable growth and productivity in arid and semiarid areas^8^. Deficit irrigation is promising tactic for saving the applied water while boosting the efficiency of water use and yields per each water unit^9–11^. Several studies revealed that the deficit irrigation strategy for different crops can save water with little yield reduction^12–14^. However, reducing irrigation water resulted in disturbance in plant physiological status^15–17^, also, drought associated lowering water hindered nutrients uptake and utilization^18,19^ associating weakness in photosynthesis, stomata opening and membrane stability^20,21^. Plants respond physiologically to water stress, which is adverse to the development and growth of plants^5^. About 83% of cultivated land is used for rainfed agriculture, which provides roughly 60% of the world’s food supply, however, the maximum yields that can be obtained from irrigation are more than double the highest yields that can be obtained from rainfed agriculture. Water availability will be the primary indicator of climate change^22^, with potentially huge and uneven effects that will have a major impact on agricultural activities. Hence, scientific management of available water resources as well as use of water saving techniques, mulching and deficit irrigation approaches of water application becomes necessary for plant growth and yield^23^.

To evolute the response of plant growth to different water volumes, previously studies have done by researchers, including on apples^24^, oranges^25^ and almonds^26^. In acid lime, maximum tree growth in respect to height and spread of plant were noted in plants irrigated with micro irrigation 100% evapotranspiration^27^. Jakhar et al.^28^ observed that highest yield of sweet orange and Kinnow were found under 1.0 volume of water through drip irrigation with plastic mulch. As an effective water saving technique and farm land management practice, soil mulching is extensively used in semi-arid and arid regions^29–31^. In earlier findings it has been reported that covering of soil with mulching is effectively improves the physical properties of soil^32^, control soil temperature^33–35^, soil fertility^36^, alter the soil microbiology^37^, regulate the rainfall infiltration^38,39^ and ultimately increase plant growth, yield and WUE^40,41^.

Water-saving techniques are crucial, particularly since many parts of the country are facing a continuous decline in the supply of irrigation water. Covering soil with mulches can keep soil moisture via shrinking the evaporation while suppressing weed plants that accompanied crop plants^42–44^. Furthermore, mulching could regulate the nutrients homeostasis in soils^45,46^. Regulation of water quantity, directly reduces water requirements and the mulches curtail direct evaporation of water, moderating soil environment and reducing the need of irrigation water. However, hardly any research related to volume of irrigation and mulch in Vertisols conditions is available on Mosambi plants. It is likely to prove useful in sustainable sweet orange cultivation regions and in other areas facing similar horticultural-ecological conditions. Keeping the efficacy of volumes of water and mulches into account, this study was undertaken to evaluate the impact of water volumes and mulches on biometric parameters of plant, Chemical contents for sweet orange leaves and soil properties of the orchard in South-Eastern humid parts of Rajasthan in India.

## Materials and methods

### Experiment location

A two-year field experiment was conducted during Aril 2018 to March 2020. The field study was carried out at the CH&F instructional farm, Jhalarapatan, Jhalawar in the newly established sweet orange orchard of cv. Mosambi which situated in the South-Eastern part of Rajasthan state (23°4 to 24°52 North-Latitude, 75°29 and 76°56 East-Longitude) in India. The experimental site falls in ‘V’ agro-climatic Zone (humid south-eastern plains). The site receives 950 mm annual effective rainfall (maximum rainfall from the South-West monsoon). During summer the temperature touches around 44–47 °C and during winter it dips down to 2 °C. The region has a high 1540 mm evaporation rate. Thus, there is still a 190 mm rainfall deficit (1540–950 mm = 190 mm). The experimental site’s soil is classified as clay soil that is commonly known as black cotton soil with a composition of 10.1% sand, 40.0% silt and 49.9% clay (Table 1).

**Table 1:** Physical and chemical properties of soil of experimental orchard.

S. no.	Soil properties	Content
1.	Clay (%)	49.9
2.	Silt (%)	40.0
3.	Sand (%)	10.1
4.	pH	7.36
5.	Electrical conductivity (d Sm^−1^)	0.45
6.	Organic carbon (%)	0.49
7.	Bulk density (mg/m^3^)	1.38
8.	Porosity (%)	47.62
9.	Water holding capacity (%)	38.68
10.	Available N (kg ha^−1^)	305.92
11.	Available P (kg ha^−1^)	25.13
12.	Available K (kg ha^−1^)	282.06

### Treatments detail and experimental design

The experimental ‘Mosambi’ sweet orange (C*itrus sinensis* Osbeck) plants were one-year-old and the orchard was planted in North- South orientation in 3240 m^2^ (90 m × 36 m) area. The experimental sweet orange orchard accommodates 90 plants with a row- plant spacing of 6 × 6 m^2^. The experiment was conducted using a Factorial Randomized Block Design (RBD) to evaluate the effects of different irrigation volumes and mulching materials on the growth, leaf nutrient status, and soil properties of Mosambi. The experimental design included two main factors: irrigation volume at three levels (100% ET_c_, 80% ET_c_, and 60% ET_c_) and five different mulching treatments (no mulch, black polythene mulch, white polythene mulch, coriander straw mulch, and dry grass mulch). Details of the treatments assessed under the study are as follows (Table 2):

**Table 2:** Treatments details.

Treatment	Details of the treatment
A. Water volumes	
I_1_	Irrigation at 100% ET_c_
I_2_	Irrigation at 80% ET_c_
I_3_	Irrigation at 60% ET_c_
B. Mulches	
M_0_	Without mulch
M_1_	Black polythene mulch
M_2_	Transparent polythene mulch
M_3_	Coriander straw mulch
M_4_	Dry grass mulch

Each treatment combination was replicated three times. The use of Factorial RBD allowed for the systematic examination of both main effects and interaction effects between the irrigation and mulching treatments, thereby providing a robust statistical analysis of the influence of these factors on the various measured parameters. The design ensured that variability within the experimental site was minimized, thus improving the accuracy and reliability of the results.

### Water volume (ETc) estimation for irrigation

**Fig. 1:**
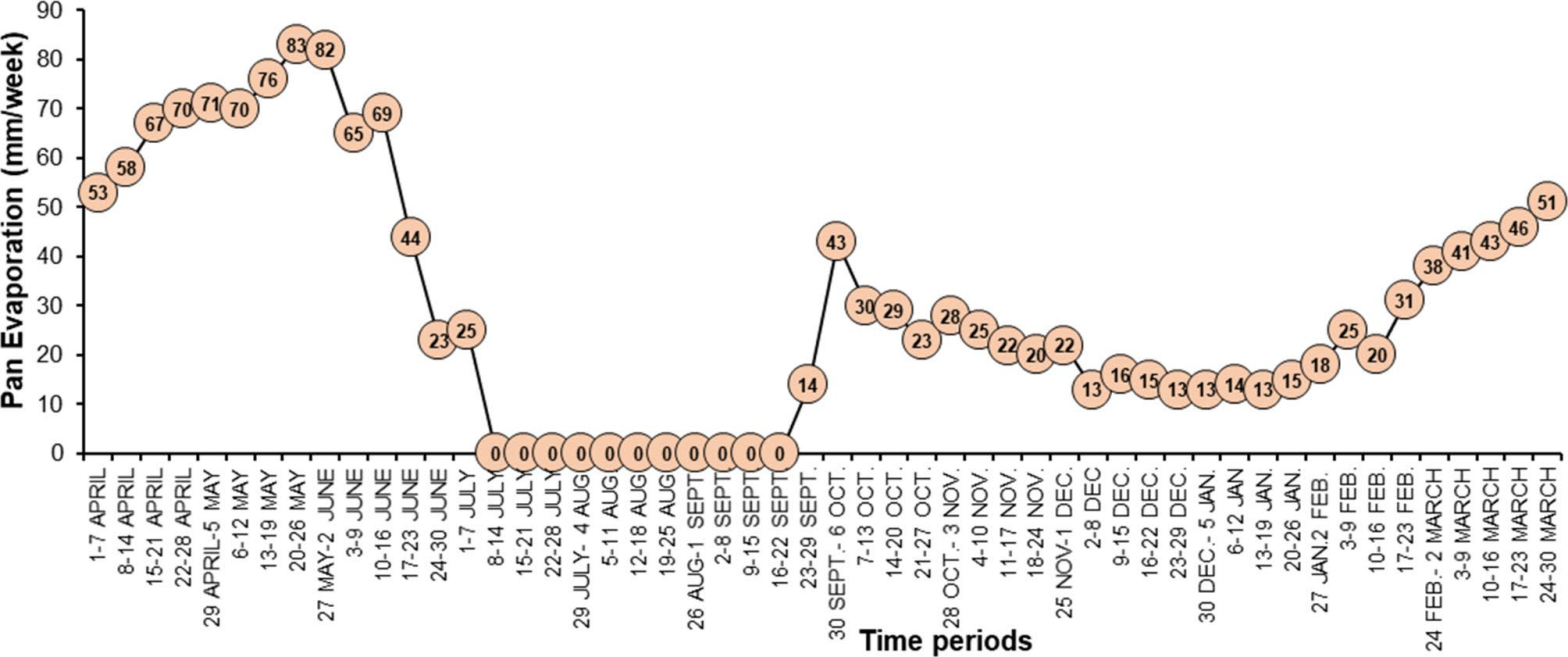
Total evaporation during a particular week for the period of experimentation (April, 2018 to March, 2020).

**Fig. 2:**
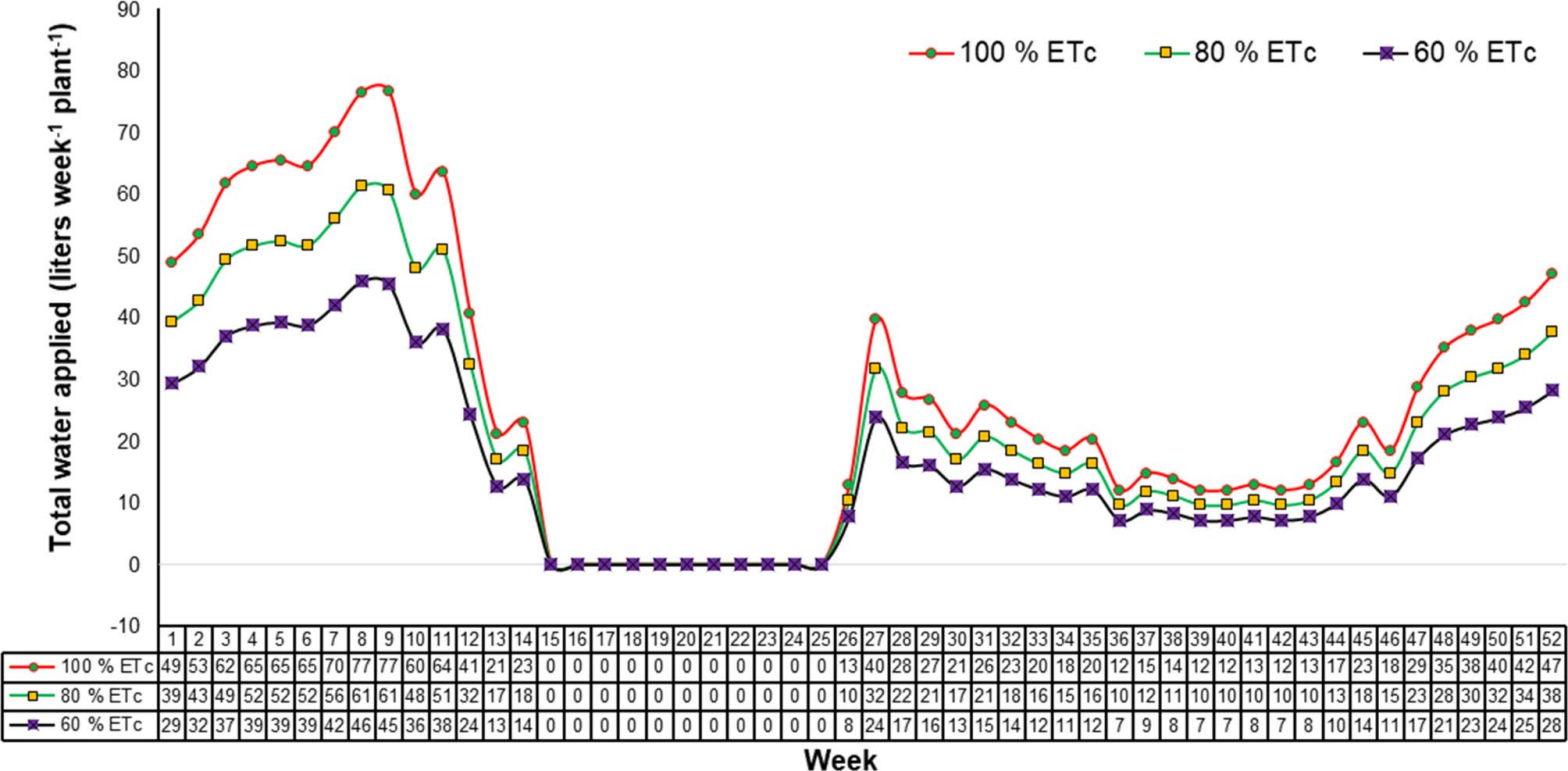
Total water applied as per evapotranspiration during a particular week for the period of experimentation (from April, 2018 to March, 2020).

The total amount of water required by the plant, known as crop evapotranspiration (ET_c_), was assessed at 100% of the Class-A pan evaporation rate^47,48^. Therefore, to determine sweet orange plant water requirements, this amount of irrigation water was used in this experiment. According to weekly pan evaporation, the amount of water applied to fully-irrigated trees was calculated (Figs. 1 and 2).

In order to irrigate the sweet orange trees according to their water requirements, Daily irrigation water was calculated through an equation of evapotranspiration^47–49^:

1 $$\:{\text{E}\text{T}}_{\text{c}}={\text{E}\text{T}}_{0}\times\:{\text{K}}_{\text{c}}\times\:\text{A}-{\text{R}}_{\text{e}}$$

2 $$\:{\text{E}\text{T}}_{0}={\text{E}}_{\text{p}}\times\:{\text{K}}_{\text{p}}$$

Where ET_0_ = reference evapotranspiration; E_p_ = Pan evaporation; K_p_ = Pan co-efficient for class A pan evaporimeter which was 0.7 and K_c_ was specific crop coefficient (K_c_ values to be 0.50 in the month of January, 0.55 in the month of February, March and November, December, 0.60 in the month of April, May and October, 0.65 in the month of June and September), 0.70 in month of July and August, A = canopy area, R_e_ = Effective rainfall in mm^50^.

Irrigation water was consistently supplied at 100% ET_c_, 80% ET_c_, and 60% ET_c_ in the respective treatments during the growth period. The irrigation period was from April first to the end of June and first October to the end of March at two days interval in each year of experiment. During growth period, watering was through drip irrigation system. Effective rainfall was noted during the rainy months. On the other hand, effective rainfall was also reported in the pre and post rainy seasons i.e., April through June and October through March, which are not monsoon months. Irrigation water supply was stopped during July to September months in view of availability of moisture from raining. Based on the amount of water to be applied to the plants in various treatments and the emitter discharge, the time of irrigation was determined.

The mulch treatments were applied during the first week of April, after recording the initial (base) growth and development parameters of the plants. Moreover, the irrigation water supply was started on April 1, 2018, and continued till March 2019, repeated during April to March 2019-20. Twenty-micron polythene mulch was used in the experiment. For the experiment, coriander straw and dry grass mulches were spread at a thickness of 10 cm. During particular year of experiment the mulches were applied only once as per treatment.

Throughout the study period, monthly data on plant growth parameters were recorded. Before the application of treatment, initially, the rootstock girth was measured using digital vernier caliper (0–450 mm, Mitutoyo), after that, it was measured at the same point at the end of every month. A measuring scale was used to get the plant’s height (in centimeters). A measuring tape was used to record the sweet orange plants’ canopy spread (East-West and North-South spread) in centimeters. Based on the cumulative increase in the initial value, the average increase in each direction was computed.

de Carvalho et al.^51^ suggested method was used to calculate the plant leaf area (cm^2^):

3 $$\:\text{A}=\text{L}\times\:\text{W}$$

Where, A = leaf area (cm^2^); L = length and W = width.

### Chlorophyll content

The chlorophyll content (mg/g) of the leaves of sweet orange plants was measured using the method recommended by Sadasivam and Manickam^52^, both before and after the experiment. One gram of mature leaves, which were three to four months old, were crushed in twenty milliliters of acetone (80%) and centrifuged at 5000 rpm for five minutes. Using a spectrophotometer, the absorbance was measured at 645 and 763 nm in comparison to the blank sample (80% acetone). To calculate the extract’s chlorophyll content, use this formula:

4 $$\:\text{T}\text{o}\text{t}\text{a}\text{l}\:\text{C}\text{h}\text{l}\text{o}\text{r}\text{o}\text{p}\text{h}\text{y}\text{l}\text{l}=20.2\:\left({\text{A}}_{645}\right)-8.02\:\left({\text{A}}_{763}\right)\times\:\frac{\text{V}}{1000\times\:\text{W}}$$

(Where, Total Chlorophyll unit is mg g^−1^; A_645_ and A_763_ are the absorbance values at 645 nm and 763 nm, respectively; V is the final volume of chlorophyll extract in acetone (80%) in milliliters and W is the fresh weight of the leaf sample in grams).

### Leaf mineral status

Sample collection and Analysis: Leaf minerals were analyzed before and after the experiment (at 1st April 2018, 2019 and after the experiment at 30th March 2019 and 2020). Throughout the study period, monthly data on plant growth parameters were recorded. As per the process suggested by Chahill et al.^53^, leaf samples were taken in order to estimate the N, P, and K status of the leaf. Leaf Nitrogen was calculated using Subbiah and Asija^54^ method. The vanado molybdo phosphoric acid technique, as recommended by Jackson^55^, was used to determine the total phosphorous from the leaf sample. Using a flame photometer, the potassium concentration of leaf tissues was calculated^55^. A muffle furnace was used to convert 1 g of powdered leaf tissue to ashes at 550 °C for up to 12 h to analyze Ca, Mg, Fe, and Zn. Using inductive coupled plasma-optical emission spectrometry, the concentrations of Ca, Mg, Fe, and Zn were measured following incineration and extraction with nitric acid (1% v/v)^56^.

### Soil mineral content

Soil mineral contents were analyzed initially and at the end of experiment. The available soil N content was calculated using the Kjeldahl method, as recommended by Salehi et al.^57^. The Olsen method was used to measure the amount of available soil phosphorus^58^. After NH_4_OAc extraction, the amount of available potassium in the soil was measured using flame photometer^29^.

### Statistical analysis

Duncan multiple range test (DMRT) and analysis of variance (ANOVA), were used to separate the means at *P* < 0.05 with the aid of WASP, to evaluate the data collected from the experiment, conducted from April 2018 to March 2020, on the effects of mulches and irrigation volumes on the growth and development of Mosambi plants. The Pearson correlation matrix was calculated for all parameters^59^. A correlation matrix is a table that shows the correlation coefficients between multiple variables in a data set^60^. It’s a statistical technique that helps you understand how variables are related to each other. A t-test (*n* = 72) was used to determine the significance of the connection.

## Result

### Phenotypic parameter

#### Height of the plant

Different water volumes and mulches significantly affect plant height. The I_1_ treatment (100% ET_c_) had the maximum percentage increase in the height of plant (28.64%) compared to all other treatments, while the I_3_ treatment (60% ET_c_) had the minimum percentage increase in plant height (19.65%). The data in Table 3 manifested the highest percent increase in plant height (30.31%) in sweet orange plants due to mulching treatment was recorded treatment M_1_ (Black polythene mulch). The minimum increment in the height of plant (19.48%) was observed due to M_0_ treatment (No mulch) at the end of experiment.

**Table 3:** Effect of irrigation volumes and mulches on plant growth parameters of sweet orange (*Citrus sinensis* Osbeck) cv. Mosambi orchard soil during two consecutive year growth period.

Treatments	Plant height (cm)	Rootstock girth (mm)	Plant spread (cm)		Leaf area
			East-west	North-south	
I_1_	28.64 ± 0.461^a^ (156.20)	36.61 ± 1.163^a^ (43.22)	63.82 ± 0.905^a^ (74.15)	63.87 ± 2.200^a^ (74.99)	2.40 ± 0.0194^a^ (28.06)
I_2_	25.50 ± 0.643^b^ (152.28)	30.85 ± 0.456^b^ (40.98)	56.25 ± 1.740^b^ (68.24)	56.07 ± 1.123^b^ (68.33)	2.30 ± 0.0201^b^ (28.94)
I_3_	19.65 ± 0.194^c^ (140.27)	23.35 ± 0.371^c^ (36.57)	46.75 ± 0.994^c^ (57.25)	46.54 ± 0.181^c^ (56.81)	2.14 ± 0.0067^c^ (24.21)
M_0_	19.48 ± 0.523^e^ (142.10)	23.47 ± 0.305^e^ (38.21)	46.45 ± 0.617^e^ (60.01)	46.31 ± 0.842^e^ (60.58)	2.11 ± 0.0032^e^ (24.59)
M_1_	30.31 ± 0.185^a^ (159.27)	37.90 ± 0.324^a^ (43.37)	67.56 ± 1.579^a^ (75.64)	67.90 ± 2.663^a^ (75.93)	2.34 ± 0.0287^a^ (28.56)
M_2_	21.61 ± 0.671^d^ (144.18)	26.26 ± 0.543^d^ (39.10)	50.08 ± 0.591^d^ (61.62)	49.82 ± 0.517^d^ (61.85)	2.17 ± 0.0087^d^ (27.65)
M_3_	24.36 ± 0.256^c^ (148.16)	30.19 ± 0.168^c^ (39.00)	54.13 ± 1.393^c^ (63.86)	53.90 ± 0.865^c^ (63.91)	2.24 ± 0.0162^c^ (28.33)
M_4_	27.24 ± 0.759^b^ (154.21)	33.52 ± 1.071^b^ (41.59)	59.82 ± 1.877^b^ (71.61)	59.55 ± 0.910^b^ (71.29)	2.28 ± 0.0203^b^ (26.23)

Note: Similar superscript letters indicate that data are statistically non-significant at 5% level of significance. I_1_: Irrigation at 100% ET_c_; I_2_: Irrigation at 80% ET_c_ and I_3_: Irrigation at 60% ET_c_, and M_0_: No mulch, M_1_: black polythene mulch, M_2_: white polythene mulch, M_3_: straw mulch and M_4_: grass mulch. Data presents mean ± standard error of three replicates (*n* = 3).

#### Rootstock girth (mm)

The influence of different volumes of water and mulches on rootstock girth of Mosambi plants was investigated and it is apparent from the experiment that different water volumes and mulches had significant effect on rootstock girth at the end of experiment. Table 3 illustrates the declining trend of rootstock growth with decreasing water volume. The full irrigation treatment (I_1_) showed the highest significant values for rootstock growth (36.61%), followed by the I_2_ treatment (30.85%) with significant differences between them, whereas, treatment I_3_ (23.35%) obtained the lowest values. The findings indicate that treatment M_1_ had the highest percent increase in rootstock girth (37.90%) among the other mulch treatments. Of course, M_0_ had the lowest percentage increase in rootstock girth (23.47%) at the end of the experiment.

#### Canopy spread (cm)

##### East-west spread (cm)

In this study, it was recorded that irrigation with different water volumes has significant effect on plant East-West spread (cm). Treatment I_1_ significantly (*p* ≤ 0.05) increases the plant East-West spread (63.82%), at the end of experiment. The minimum increase (46.75%) was noted in I_3_ treatment. A significant (*p* ≤ 0.05) difference due to different mulching material on plant East-West spread was recorded during the experimentation. As shown in Table 3, it is clarified that significantly maximum percent increase (67.56%) was there in M_1_ treatment. While the treatment M_0_ treatment showed minimum increase in plant East-West spread (46.45%).

##### North-south spread (cm)

Water volumes and mulches were significantly influenced the North-South spread of Mosambi plants (Table 3). Treatment I_1_ showed the highest increase in North-South spread (63.87%) compared to other treatments and control, with a significant (*p* ≤ 0.05) difference. However, it was recorded minimum (46.54%) in treatment I_3_. Maximum increase in plant North-South spread (67.90%) due to different mulching was there in M_1_ treatment at the end of experiment. The increase in plant North-South spread (46.31%) was recorded minimum in M_0_ treatment.

#### Leaf area

In both years of study, the increment in leaf area readily responded to different irrigation volumes. Treatment I_1_ had the highest values for plant leaf area (2.40%) and found significantly superior over other treatments statistically (Table 3). However, minimum increase in leaf area at the end of experiment (2.14%) was recorded under I_3_ treatment. The data analysis showed that treatment M_1_ had the highest percentage increase in leaf area (2.34%) among the treatments which was found to be significantly higher over other treatments. While the minimum percent increase (2.11%) was noted in treatment M_0_ (Table 3).

### Leaf chemical contents of sweet orange

**Fig. 3:**
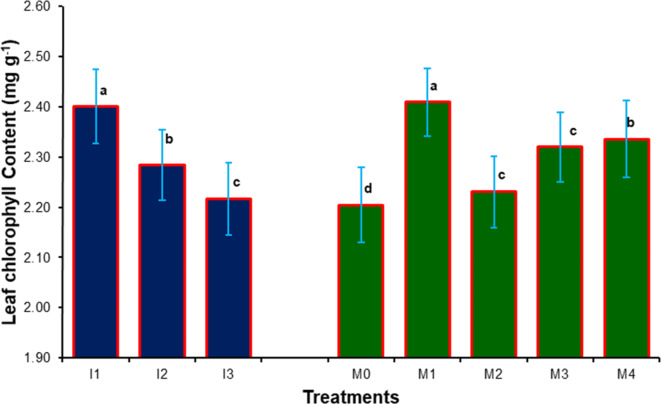
Effect of water volumes and mulches on chlorophyll content of sweet orange (*Citrus sinensis* Osbeck) cv. Mosambi during two-year growth period (April, 2018 to March, 2020).

**Fig. 4:**
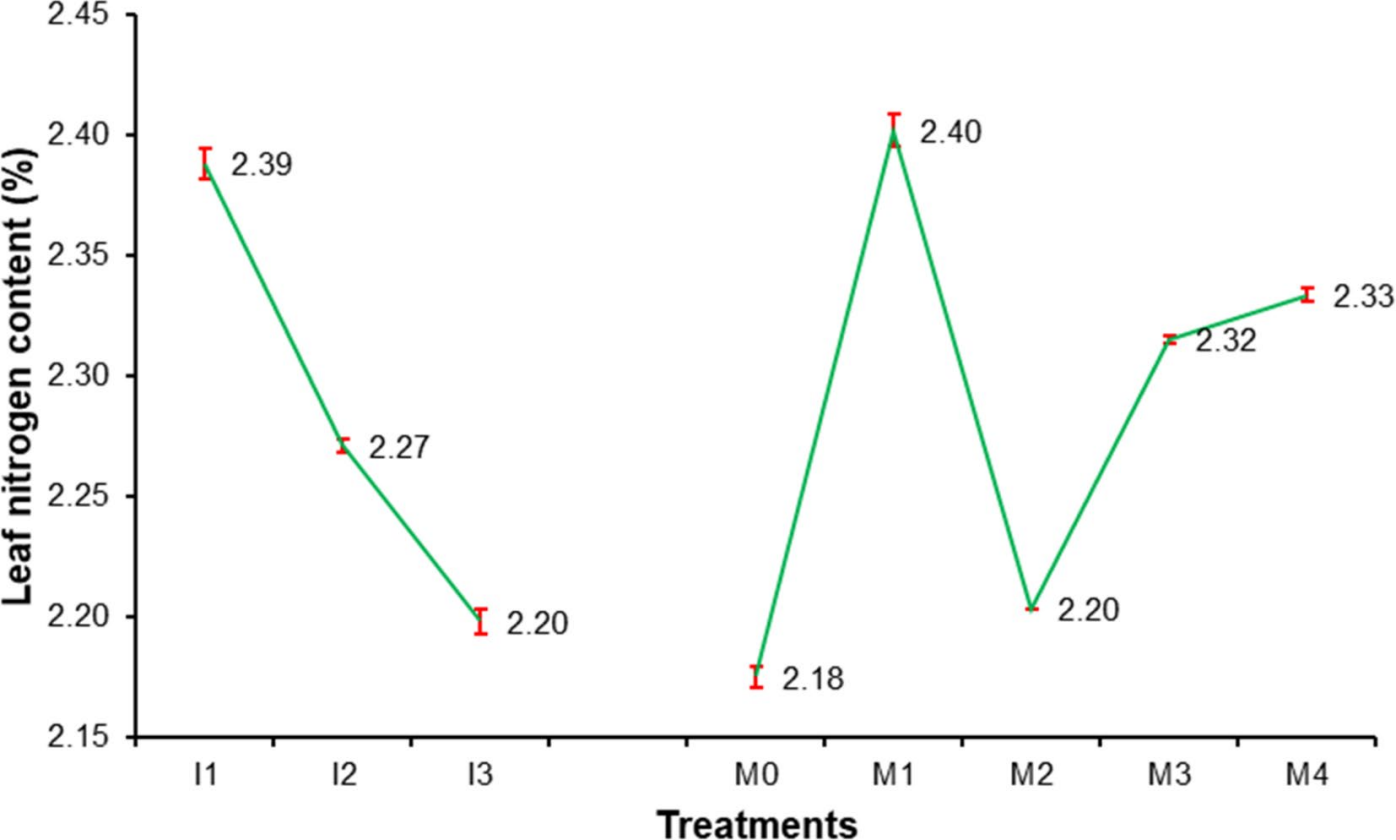
Effect of water volumes and mulches on leaf nitrogen content of sweet orange (Citrus sinensis Osbeck) cv. Mosambi during two-year growth period (April, 2018 to March, 2020).

**Fig. 5:**
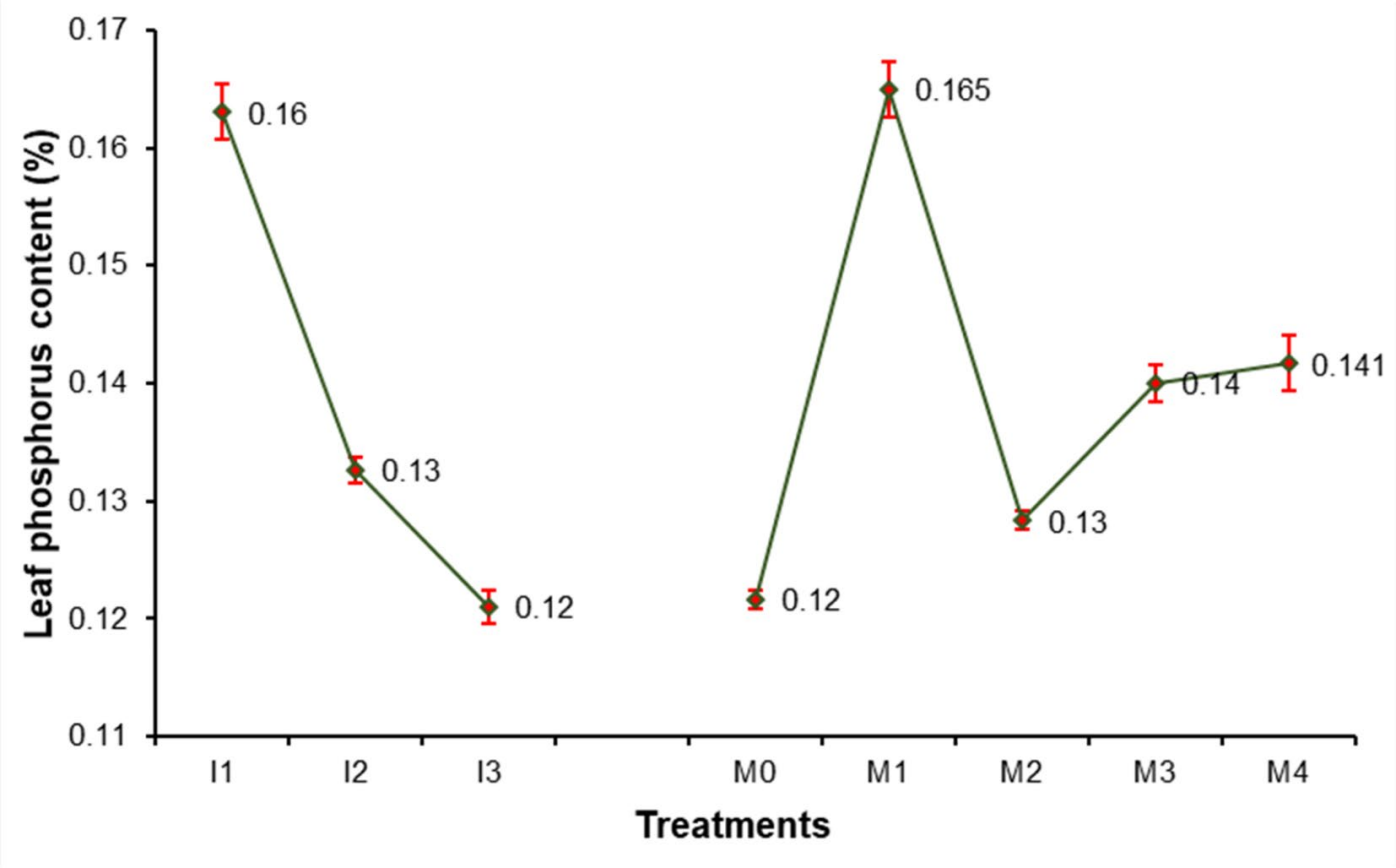
Effect of water volumes and mulches on leaf Phosphorus content of sweet orange (Citrus sinensis Osbeck) cv. Mosambi during two-year growth period (April, 2018 to March, 2020).

**Fig. 6:**
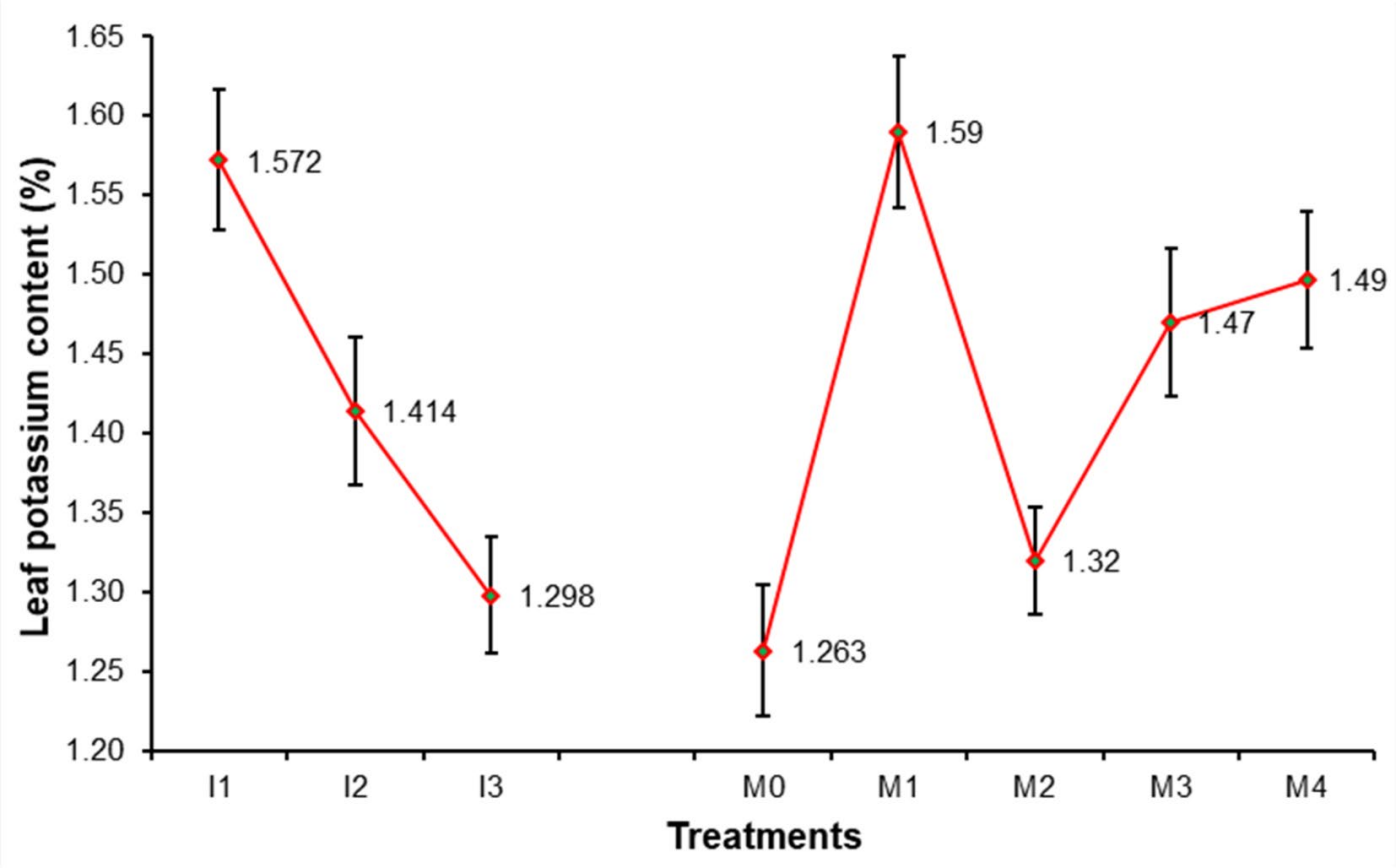
Effect of water volumes and mulches on leaf Potassium content of sweet orange (Citrus sinensis Osbeck) cv. Mosambi during two-year growth period (April, 2018 to March, 2020).

**Fig. 7:**
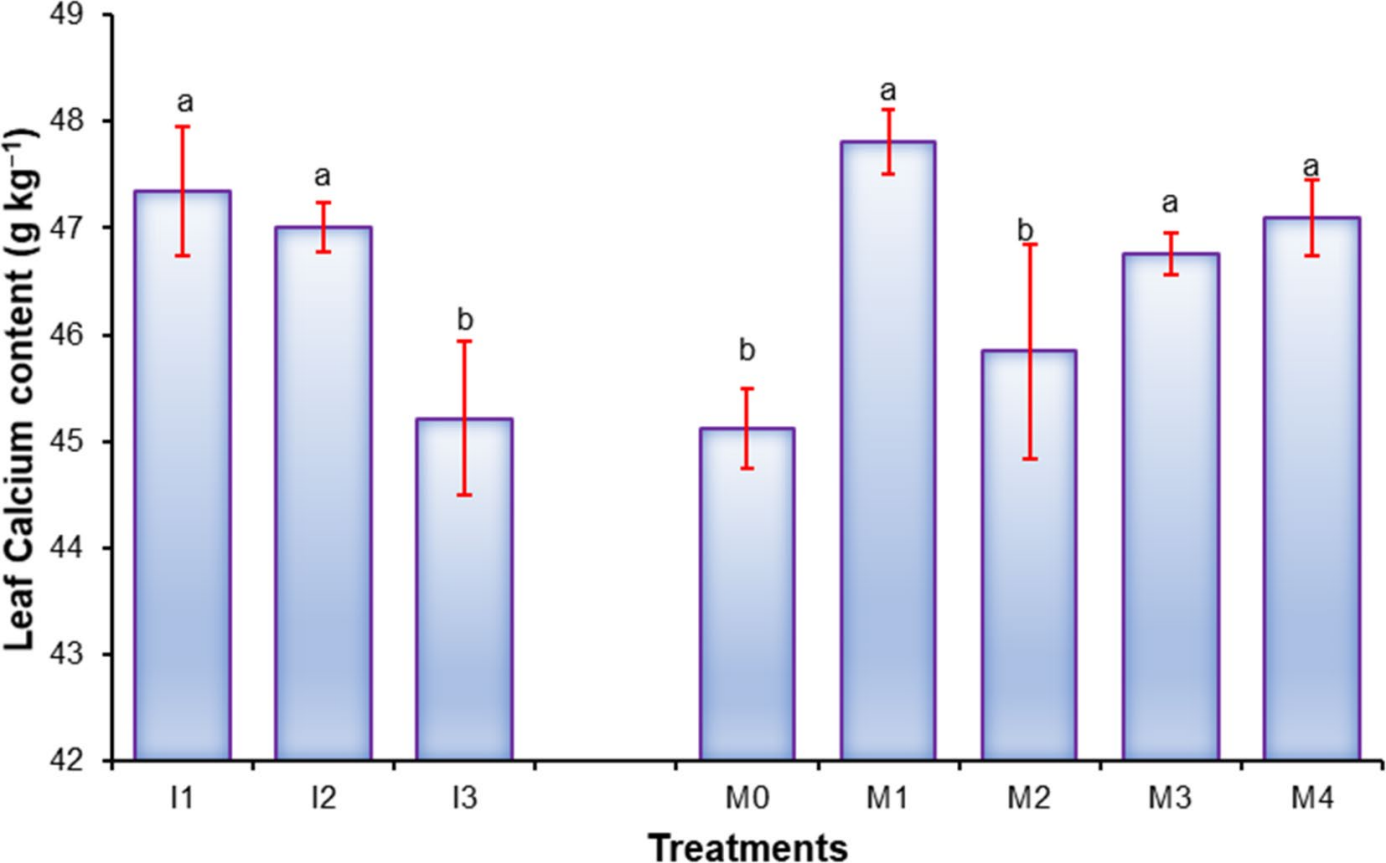
Effect of water volumes and mulches on leaf Calcium content of sweet orange (Citrus sinensis Osbeck) cv. Mosambi during two-year growth period (April, 2018 to March, 2020).

**Fig. 8:**
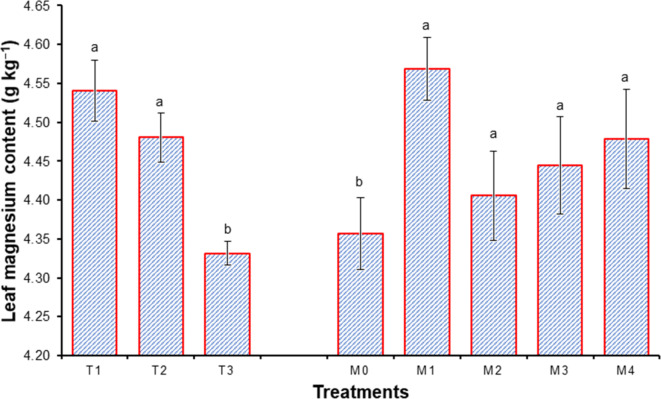
Effect of water volumes and mulches on leaf Magnesium content of sweet orange (Citrus sinensis Osbeck) cv. Mosambi during two-year growth period (April, 2018 to March, 2020).

**Fig. 9:**
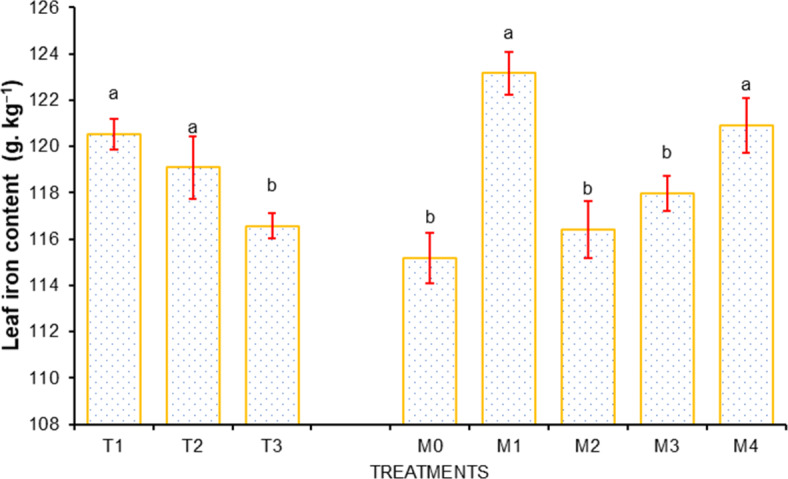
Effect of water volumes and mulches on leaf Iron content of sweet orange (Citrus sinensis Osbeck) cv. Mosambi during two-year growth period (April, 2018 to March, 2020).

**Fig. 10:**
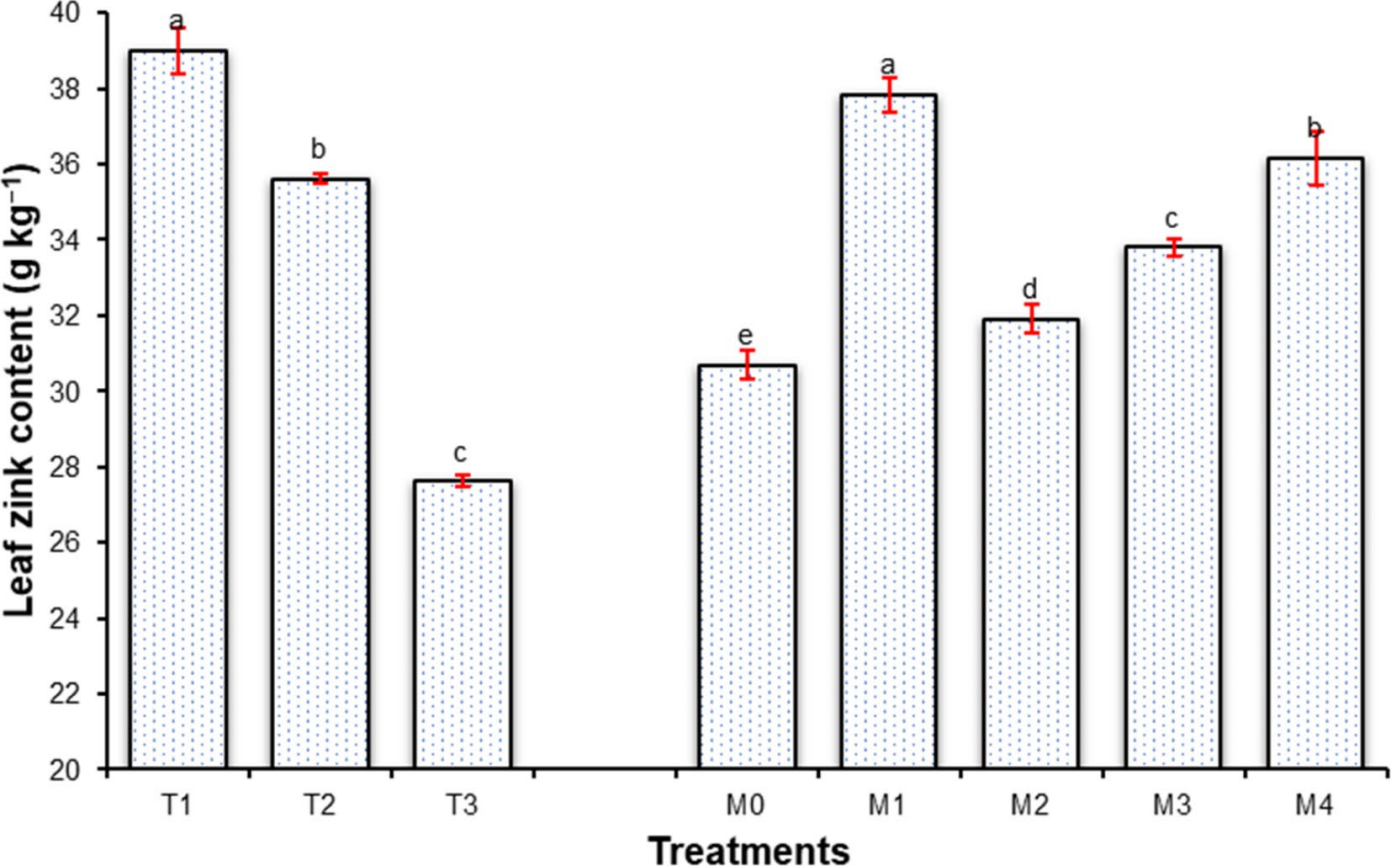
Effect of water volumes and mulches on leaf Zink content of sweet orange (Citrus sinensis Osbeck) cv. Mosambi during two-year growth period (April, 2018 to March, 2020).

Data presented in Figs. 3, 4, 5, 6, 7, 8, 9 and 10 reveal the impact of water volumes and mulches on chemical contents for Mosambi leaves (chlorophyll content and mineral elements: N, P, K, Ca, Mg, Zn and Fe). The findings showed that the photosynthetic pigments and mineral content of sweet orange leaves were significantly decreased as irrigation water was reduced from 100% ET_c_ to 60% ET_c_.

#### Leaf chlorophyll content

It is reflected from the data (Fig. 3) that leaf chlorophyll content of the plants significantly affected by water volumes and mulch treatments. The maximum leaf chlorophyll content of plant (2.40 mg g^[-1^) was noted in a I_1_ treatment compared to other treatments. However, minimum leaf chlorophyll content of the plants (2.22 mg g^[-1^) was recorded in I_3_ treatment. It is exhibited from the data that maximum increase in leaf chlorophyll content (2.41 mg g^[-1^) due to mulching treatments was there in treatment M_1_ among all the mulch treatments. The leaf chlorophyll content of plant was recorded minimum (2.20 mg g^[-1^) in M_0_ treatment.

#### Leaf mineral content

It appeared from the data that I_1_ treatment which was found significantly superior among other treatments with 2.39% increase in leaf N content due to water volumes. Whereas, it was noted minimum in treatment I_3_ (2.20%) (Fig. 4). The data showed that there was significant increase in leaf N content due to mulches. It was found maximum (2.40%) in M_1_ treatment. At the end of experiment, a minimum increase (2.18%) in leaf N content was observed in M_0_ treatment.

Maximum leaf P content due to different water volume treatments (0.16%) was recorded in I_1_ treatment compared to other treatments, whereas, it was noted minimum (0.12%) in treatment I_3_ (Fig. 5). The results clearly show that the M_1_ treatment had the highest leaf P content (0.17%) due to mulching. The minimum increase in leaf P content (0.12%) was recorded in treatment M_0_.

From Fig. 6, it is reflected that maximum leaf K content due to different water volume treatments (1.57%) was recorded in I_1_ treatment, which was significantly higher over other treatments, whereas, it was found minimum (1.29%) in treatment I_3_. It is clear from the data that maximum leaf K content (1.59%) due to different mulching treatments was recorded in M_1_ treatment. The minimum increase in leaf K content (1.26%) was recorded in treatment M_0_.

Moreover, the highest concentrations of Ca, Mg, Fe, and Zn, due to water volume treatment were recorded for I_1_ treatment (Ca, 47.35 g kg^−1^; Mg, 4.54 120.52 g kg^−1^; Fe, 120.52 g kg^−1^ and Zn, 39.00 g kg^−1^), followed by I_2_ treatment (Ca, 47.01 g kg^−1^; Mg, 4.48 g kg^−1^ and Fe, 119.09 g kg^−1^), and the minimum were recorded for I_3_ treatment (Ca, 45.21 g kg^−1^; Mg 4.33 g kg^−1^; Fe, 116.57 g kg^−1^ and Zn 27.62 g kg^−1^), presented in Figs. 7, 8, 9 and 10.

Likewise, the maximum concentrations of Ca, Mg, Fe, and Zn, due to mulch treatments were found in M_1_ treatment (Ca, 47.80 g.kg^−1^; Mg, 4.54 120.52 g.kg^−1^; Fe, 123.15 g.kg^−1^ and Zn, 37.84 g.kg^−1^), and the minimum were recorded for I_3_ treatment (Ca, 45.11 g.kg^−1^; Mg 4.36 g.kg^−1^ ; Fe, 115.19 g.kg^−1^ and Zn, 30.69 g.kg^−1^) Depicted through Figs. 7, 8, 9 and 10.

### Soil mineral parameters

#### Available soil N (kg ha^-1^)

Table 4 illustrates the available soil N (kg ha^−1^) data pertaining to sweet orange plants cv. Mosambi orchard soil mulches and water volumes. The data showed that the maximum increase in available soil N (kg ha^−1^) due to water volume treatments (314.11 kg ha^−1^) was recorded under treatment I_1_, which was significantly superior over all other treatments. However, minimum increase in available soil N (296.09 kg ha^−1^) was recorded under I_3_ treatment. It is reflected from the data presented in Table 4 that there was maximum increase in available soil N due to different mulches (315.70 kg ha^−1^) in M_1_ treatment, which was significantly superior over all other treatments. The minimum increase in available soil N (295.37 kg ha^−1^) was recorded in M_0_ treatment.

**Table 4:** Effect of water volumes and mulches on soil parameters of sweet orange (Citrus sinensis Osbeck) cv. Mosambi during two years growth period (April, 2018 to March, 2020).

Treatment	Soil *N* (kg ha^-1^)	Soil *P* (kg ha^-1^)	Soil K (kg ha^-1^)
Initial values	305.92	25.13	282.06
I_1_	314.11 ± 8.87^a^	28.93 ± 0.58^a^	291.53 ± 8.34^a^
I_2_	304.87 ± 9.58^b^	24.82 ± 0.38^b^	281.99 ± 9.25^b^
I_3_	296.09 ± 9.80^c^	20.76 ± 0.59^c^	272.27 ± 8.93^c^
M_0_	295.37 ± 8.76^d^	22.09 ± 0.49^e^	271.68 ± 8.91^d^
M_1_	315.70 ± 10.24^a^	29.38 ± 0.571^a^	293.19 ± 8.06^a^
M_2_	297.98 ± 8.28^c^	22.86 ± 0.12^d^	273.90 ± 8.51^c^
M_3_	307.18 ± 9.46^b^	24.39 ± 0.79^c^	284.79 ± 9.34^b^
M_4_	308.88 ± 10.35^b^	25.45 ± 0.72^b^	286.10 ± 9.34^b^

Note: Similar superscript letters indicate that data are statistically non-significant at 5% level of significance. I_1_: Irrigation at 100% ET_c_; I_2_: Irrigation at 80% ET_c_ and I_3_: Irrigation at 60% ET_c_, and M_0_: No mulch, M_1_: black polythene mulch, M_2_: white polythene mulch, M_3_: straw mulch and M_4_: grass mulch. Data presents mean ± standard error of three replicates (*n* = 3).

#### Available soil P (kg ha^-1^)

The available phosphorus (kg ha^−1^) diminished significantly with decreasing water volume from 100% ET_c_ to 60% ET_c_ (Table 4). Nevertheless, significantly (*p* ≤ 0.05) maximum available soil P (kg ha^−1^) (28.93 kg ha^−1^) at the end of experiment was recorded in treatment I_1_ compared to all other treatments, on other hand, Treatment I_3_ showed lowest value of available soil P (kg ha^−1^) (20.76 kg ha^−1^) in soil. At the end of experiment, the maximum available soil P (29.38 kg ha^−1^) due to mulching treatments was there in treatment M_1_. However, treatment M_0_ showed minimum (22.09 kg ha^−1^) available P content in orchard soil.

#### Available soil K (kg ha^-1^)

The availability of K (kg ha^−1^) in orchard soil was significantly (*p* ≤ 0.05) affected by water volumes and mulches at the end of study. The maximum available soil K (291.53 kg ha^−1^) was recorded under treatment I_1_, whereas, it was found minimum (272.27 kg ha^−1^) in treatment I_3_. Furthermore, maximum available soil K (293.19 kg ha^−1^) due to mulching was recorded in treatment M_1_. The minimum increase in available soil K (271.68 kg ha^−1^) was recorded in treatment M_0_ at the end of experiment (Table 4).

**Table 5:**
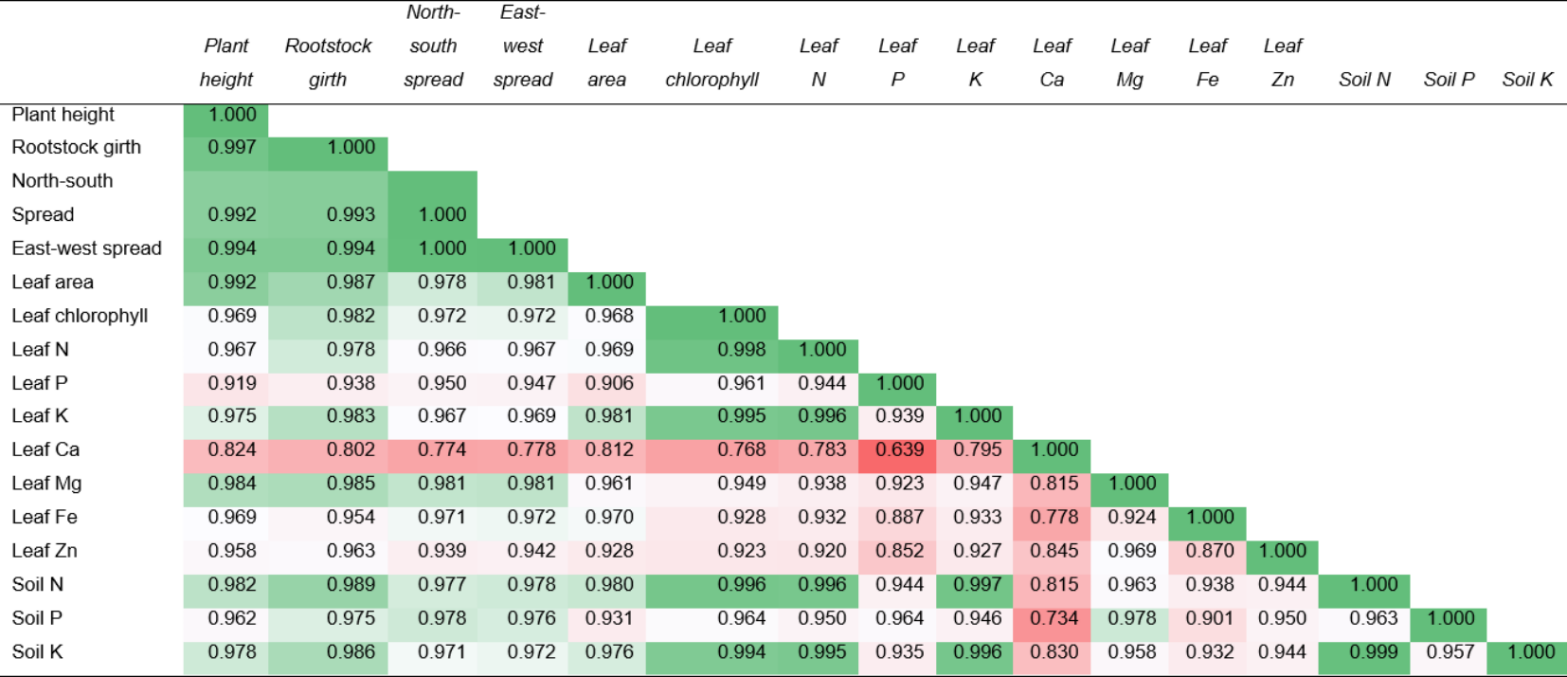
Correlation between plant growth, leaf chemicals and soil parameters of Sweet orange plants cv Mosambi.

Different colours represent the correlation either strongly positive or positive. The value in dark green colour shows the strongly positive correlation, between different plant and soil parameters.

### Correlation matrix

Table 5 illustrates a correlation matrix indicating the degree of linear correlation among plant height and all other characteristics (Rootstock girth, canopy spread, leaf area, leaf chlorophyll, leaf macronutrients and micronutrients, and soil macronutrients). The results indicate a strong positive correlation across most variables, with dark green values denoting the strongest correlations. Plant height, rootstock girth, and canopy spread (both North-South and East-West) show nearly perfect correlations with each other (values above 0.99), suggesting that these growth parameters are closely linked. Leaf area also correlates strongly with growth parameters, particularly with plant height and rootstock girth.

Leaf chlorophyll content shows a very strong correlation with leaf nitrogen (0.998) and also has significant correlations with other growth and nutrient parameters. The soil nutrients, particularly nitrogen and potassium, exhibit strong correlations with most of the plant and leaf parameters, indicating that soil fertility directly influences plant growth and leaf chemical composition. Specifically, soil nitrogen shows almost perfect correlations with leaf nitrogen (0.996) and leaf potassium (0.997), underscoring the critical role of these nutrients in supporting plant health and growth. Leaf calcium and magnesium have relatively weaker correlations with the other parameters, suggesting that while important, their influence may not be as pronounced as that of other nutrients. Overall, the data highlight the intricate relationships between soil fertility, leaf chemistry, and plant growth, with certain nutrients playing pivotal roles in the overall health and development of the sweet orange plants.

## Discussions

The present study demonstrated that both irrigation volumes and mulching significantly influence the growth, leaf nutrient status, and soil mineral content of Sweet Orange (Citrus sinensis) cv. Mosambi. The findings revealed that the treatment with 100% ET_c_ irrigation (I_1_) and black polythene mulch (M_1_) consistently produced superior outcomes in plant growth parameters, leaf chemical contents, and soil nutrient availability.

The positive impact of full irrigation (I_1_) on plant height, rootstock girth, and canopy spread can be attributed to the adequate water supply, which supports optimal physiological processes, such as cell expansion, nutrient uptake, and photosynthesis. This is consistent with previous studies that have shown enhanced vegetative growth under sufficient irrigation in various fruit crops​^61^. Water stress, as observed in the 60% ET_c_ treatment (I_3_), significantly reduced these growth parameters, indicating the detrimental effects of limited water availability on plant development. Similarly, black polythene mulch (M_1_) was found to be the most effective mulching treatment, leading to significant improvements in plant height, canopy spread, leaf area, and chlorophyll content. The benefits of black polythene mulch can be attributed to its ability to conserve soil moisture, regulate soil temperature, suppress weed growth, and enhance soil structure. These factors create a favorable microenvironment around the root zone, facilitating better water and nutrient uptake, which in turn promotes higher growth and productivity.

Better growth of shoot parameters under I_1_ treatment over other treatments might be due to the better effectivity of this treatment in supply of adequate quantity of water which perhaps led to increase in mesophyll cells mitotic activity in the growing zone of plant which further might enhanced the zone of cell division^62^. From a study Tiwari et al.^63^ mentioned that, there might have been improved cell expansion triggered by mass flow dependent better movement of mineral nutrients under this treatment, which in turn manifested comparatively higher growth under the influence of I_1_ treatment. The findings are in line with the experiment conducted by Me et al.^64^ and El-Shawadfy^65^, which suggested that increasing the level of irrigation from 60 to 100% ET_c_ significantly enhanced plant vegetative growth. This might be due to the role of water in increasing the uptake of mineral elements from the soil, which is followed by the translocation of photosynthetic assimilates, which in turn reflects increases in plant growth^66^. Moreover, plants undergo a variety of physiologic and biochemical changes such as weakens the membrane structure and permeability, protein structure and function, which leads to cell death because of water stress^67,68^. The current findings are in line with the results presented in the studies on cucumber by Parkash et al.^69^, sweet orange by Tiwari et al.^63^, and acid lime by Goramnagar et al.^27^.

Comparably, the increased growth of plants under M1 treatment might be attributed to improvement in water conservation, improvement of soil structure turning it loose and well aerated, minimizing moisture loss from the soil due to evaporation, minimizing soil salinity^70^ due to the formation of humic acid^63^, regulating soil temperature^71^, completely suppressing weed development^72^ due to the absence of light penetration, reducing the plant weed competition, along with higher absorption of nutrients under a moderated microenvironment^73^, amid various other advantages. It might be a useful method for increasing beneficial soil microbes by improving its microclimate in the rhizosphere, increasing the mineral uptake in plants from the surface of soil and stimulating surface roots^74^ that could lead to a higher increase in shoot attributes in the treatment M_1_. Haneef et al.^75^ in pomegranates and Adnan et al.^76^ in strawberries also noted similar observations of beneficial results with the application of black polythene mulch.

The enhanced leaf nutrient content, particularly nitrogen (N), phosphorus (P), potassium (K), calcium (Ca), magnesium (Mg), iron (Fe), and zinc (Zn) in plants under I_1_ and M_1_ treatments, underscores the importance of adequate irrigation and effective mulching in maintaining nutrient balance within the plant. The improved nutrient status likely resulted from the higher availability of nutrients in the soil, reduced nutrient leaching, and better root function under optimal soil moisture conditions^77–79^. This aligns with the findings of other studies where sufficient irrigation and mulching led to higher nutrient uptake and improved plant health​^46,80^. Moreover, the correlation analysis revealed strong positive relationships between plant growth parameters and leaf nutrient contents, indicating that the enhancement of one aspect positively influences others. For instance, the high correlation between leaf chlorophyll content and leaf nitrogen highlights the direct role of nitrogen in chlorophyll synthesis, which is crucial for photosynthesis and overall plant growth.

The findings agree with study conducted by Ghorbanli et al.^81^ and Me et al.^64^ as they noted that chlorophyll content and leaf nutrient declined in mild and severe water stress conditions in tomato. The better improvement in leaf nutrient content under treatment I_1_ over other treatments could be attributed due to the presence of enough water in the soil, which could enhance the plant’s hydraulic process, act as a transporter of nutrients from the soil to the green plant tissues, and function as a strong agent of thermo-regulation^82^. As a result, there was a noticeable rise in stomatal conductance and transpiration^83^, which promotes CO_2_ to enter the leaf and enhance photosynthetic rate (favoured CO_2_ assimilation)^84^. It appears that increased CO_2_ assimilation via impacts on cell division and elongation may have increased leaf area and other characteristics. Al-Desouki et al.^85^ have reported an association between sufficient water supply and higher chlorophyll content in leaves. Since low water supply declined the plant pigments and leaf water content, crop growth was reduced^86–88^. Due to drought, imbalance in osmolytes and nutrients with lipid peroxidation of plant cells were assessed^89–91^. Whereas, drought stress decreases photosynthetic rate which might be due to reduction in stomatal conductance triggered by increased abscisic acid content in plant leaves^92^. The results shown above are like previous studies reported in Nagpur Mandarin by Panigrahi and Srivastava^93^ and in Apple v. Anna by Soliman et al.^94^.

Likewise, higher leaf nutrient content in M_1_ treatment might be explained in the light of fact of enhancement in physical and chemical properties of soil through comparatively congenial environment in the root zone, modification in the radiation of the surface and thus created better environment for plant growth. Higher chlorophyll content in this treatment might be due to the higher nitrogen accumulation in plants that perhaps subsequently helped in production of chlorophyll as quoted by Sarkar et al.^95^. Reduction of nutrient leaching and volatilization, leads to increased nutrient diffusion into the plant root zone may be responsible for of the treatment’s higher leaf nutritional content. Increased leaf nutrition, particularly nitrogen content, has been correlated to increased leaf development through a chain of activities including conversion of synthesized carbohydrates into amino acid and protein in the leaf^93,96^.

A low redox potential caused by low oxygen concentration, the higher solubility of iron in its reduced form (Fe_3_^+^ to Fe^+^), and other micronutrients in soil might be the cause of the improved availability of micronutrients in I_1_ treatment^97^. Improved micronutrient concentrations in plant leaves under the fully irrigated plants were also noticed by Panigrahi and Srivastava^93^ in Kinnow, Marathe et al.^98^ in pomegranates and Jat et al.^99^ in guava. Different mulches also influenced leaf micronutrient nutrient content (Ca, Mg, Zn, and Fe) during the study. The maximum concentrations of the micronutrients were recorded in plants under M_1_ treatment, whereas it was recorded minimum in M_0_ treatment (un-mulched plants). The findings are in conformity with^100^ for the guava cv. Lalit and Jat et al.^99^ for guava, which similarly found higher micronutrient concentrations in guava under mulched conditions.

In terms of soil nutrient content, the study found that soil nitrogen, phosphorus, and potassium levels were significantly higher in the I_1_ and M_1_ treatments. This suggests that adequate irrigation and effective mulching not only benefit the plant directly but also improve soil fertility by maintaining higher nutrient concentrations in the soil. This can be attributed to reduced nutrient loss through leaching and the enhanced activity of soil microorganisms under these conditions​. The better enhancement in chemical parameters of soil in I_1_ treatment might be attributed to better soil micro-climate that favours soil micro-organisms in the presence of optimum soil moisture which improves soil physical structure (bulk density, porosity)^101,102^. The better availability of soil nitrogen content in this treatment might be due to the favorable soil-moisture environment by adequate volume of water supply, thus leading to less mobility and losses of nitrogen by leaching and deep percolation^103^. The findings of Fanish and Muthukrishnan^104^ agreement with the above findings. By regulating the radiation budget, changing the physical properties of the soil, and retaining soil moisture, plastic mulch directly impacts the microclimate of the soil surrounding the plant^105^. The higher available soil: N, P and K content in soil covered with black polythene mulch might be due to changed soil moisture content, soil microbial communities and redox potential. Black polythene mulch enhances soil temperature. Increasing in temperature of soil solution, the nutrient concentration such as phosphorus and potassium enhanced in the rhizosphere. The basal ATPase, phosphorus, and potassium activities in the presence of K ions were reported associated with the Root levels of these cations^106^. Djaman et al.^107^ and Das and Dutta^108^ have provided similar explanations for improved soil parameters (physical and chemical) with the application of water at 100% ET_c_ and black polythene mulch.

In conclusion, the study demonstrates that the combination of 100% ET_c_ irrigation and black polythene mulch is highly effective in promoting the growth, nutrient status, and overall health of Sweet Orange cv. Mosambi plants. These findings have practical implications for improving the management of water and soil resources in citrus orchards, particularly in regions facing water scarcity. Future research could explore the long-term effects of these treatments on fruit yield and quality to further validate their benefits.

## Conclusion

Water volumes and mulches play crucial roles in determining the growth, health, and productivity of sweet orange trees, as well as influencing the nutrient status of leaves and soil mineral content in orchards. In conclusion, during the two years of investigation, treatment I_1_ (100% ET_c_) and treatment M_1_ (black polythene mulch) substantially enhanced the growth characteristics and leaf parameters. Additionally, in both years, the same treatments increased the level of chlorophyll, N, P, K, Ca, Mg, Fe, and Zn in the leaves as well as the NKP content of the soil. Hence, the utilization of an adequate volume of water and black polythene mulching proved to be best and could be used for higher growth and productivity of sweet orange (Mosambi) crop.

Mulching involves covering the soil surface around the base of the orange trees with organic materials like wood chips, straw, or compost. Mulches offer several benefits: Moisture Retention, Weed Suppression, Temperature Regulation, Soil Health etc. In contrast, proper management of water volumes and mulches is essential for promoting the growth, health, and productivity of sweet orange trees, while also enhancing the nutrient status of leaves and soil mineral content in orchards. By ensuring adequate moisture, nutrient availability, and soil health, growers can optimize orange production and quality while minimizing environmental impact.

## Data Availability

The datasets used and/or analyzed during the current study are available from the corresponding author on reasonable request.
